# Supplementary material for: A local translation program regulates centriole amplification in the airway epithelium
Source: Sci Rep. 2023 May 1;13:7090. doi: 10.1038/s41598-023-34365-8 (PMC10151349; doi:10.1038/s41598-023-34365-8)
Supplement: Supplementary file 1 — Supplementary Information. [file 41598_2023_34365_MOESM1_ESM.pptx]

## Slide 1
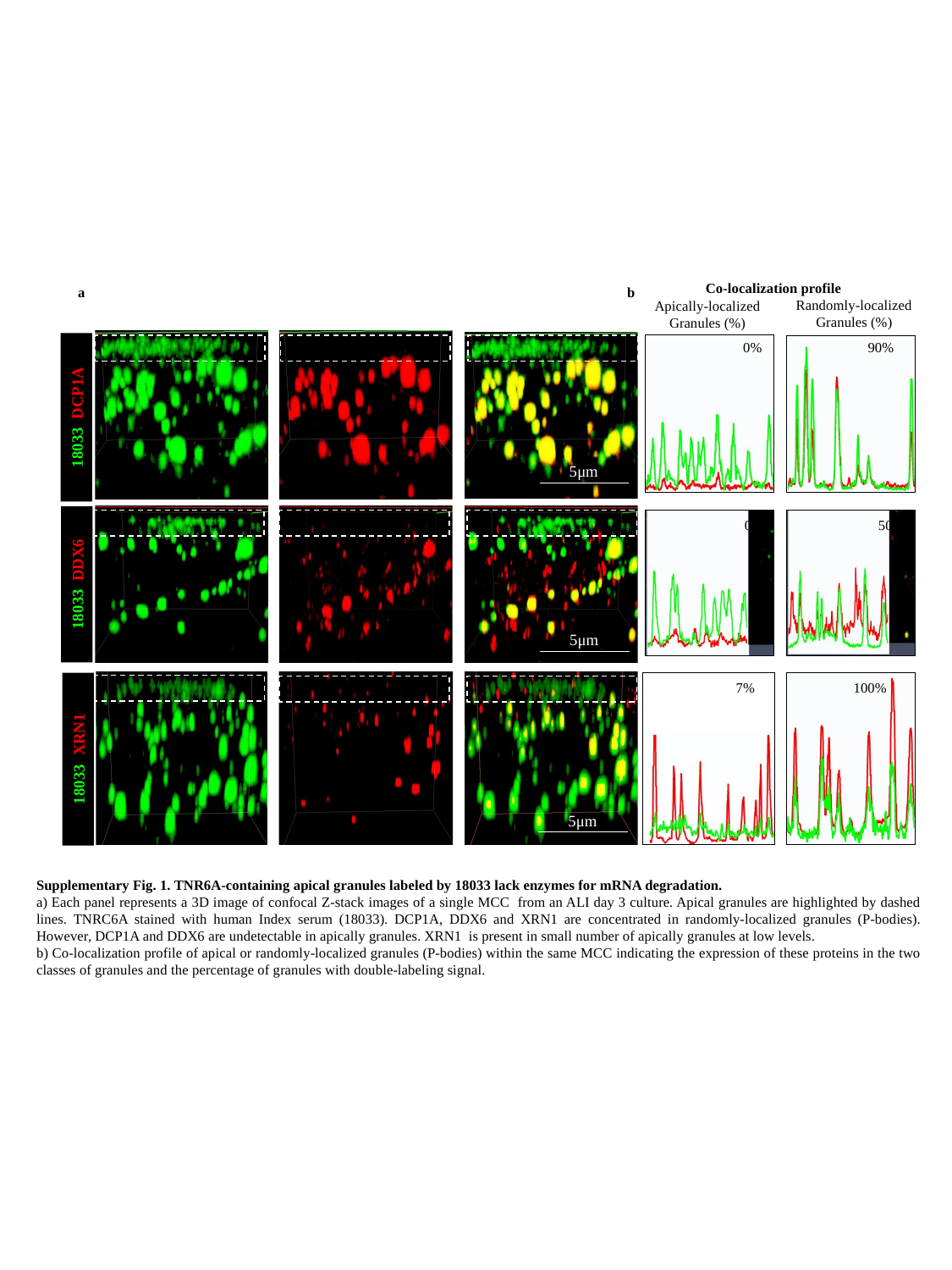

Co-localization profile
Randomly-localized
Granules (%)
Apically-localized
Granules (%)
0%
90%
0%
50%
7%
100%
a
b
18033 DCP1A
5μm
18033 DDX6
5μm
18033 XRN1
5μm
Supplementary Fig. 1. TNR6A-containing apical granules labeled by 18033 lack enzymes for mRNA degradation.
a) Each panel represents a 3D image of confocal Z-stack images of a single MCC from an ALI day 3 culture. Apical granules are highlighted by dashed lines. TNRC6A stained with human Index serum (18033). DCP1A, DDX6 and XRN1 are concentrated in randomly-localized granules (P-bodies). However, DCP1A and DDX6 are undetectable in apically granules. XRN1 is present in small number of apically granules at low levels.
b) Co-localization profile of apical or randomly-localized granules (P-bodies) within the same MCC indicating the expression of these proteins in the two classes of granules and the percentage of granules with double-labeling signal.

## Slide 2
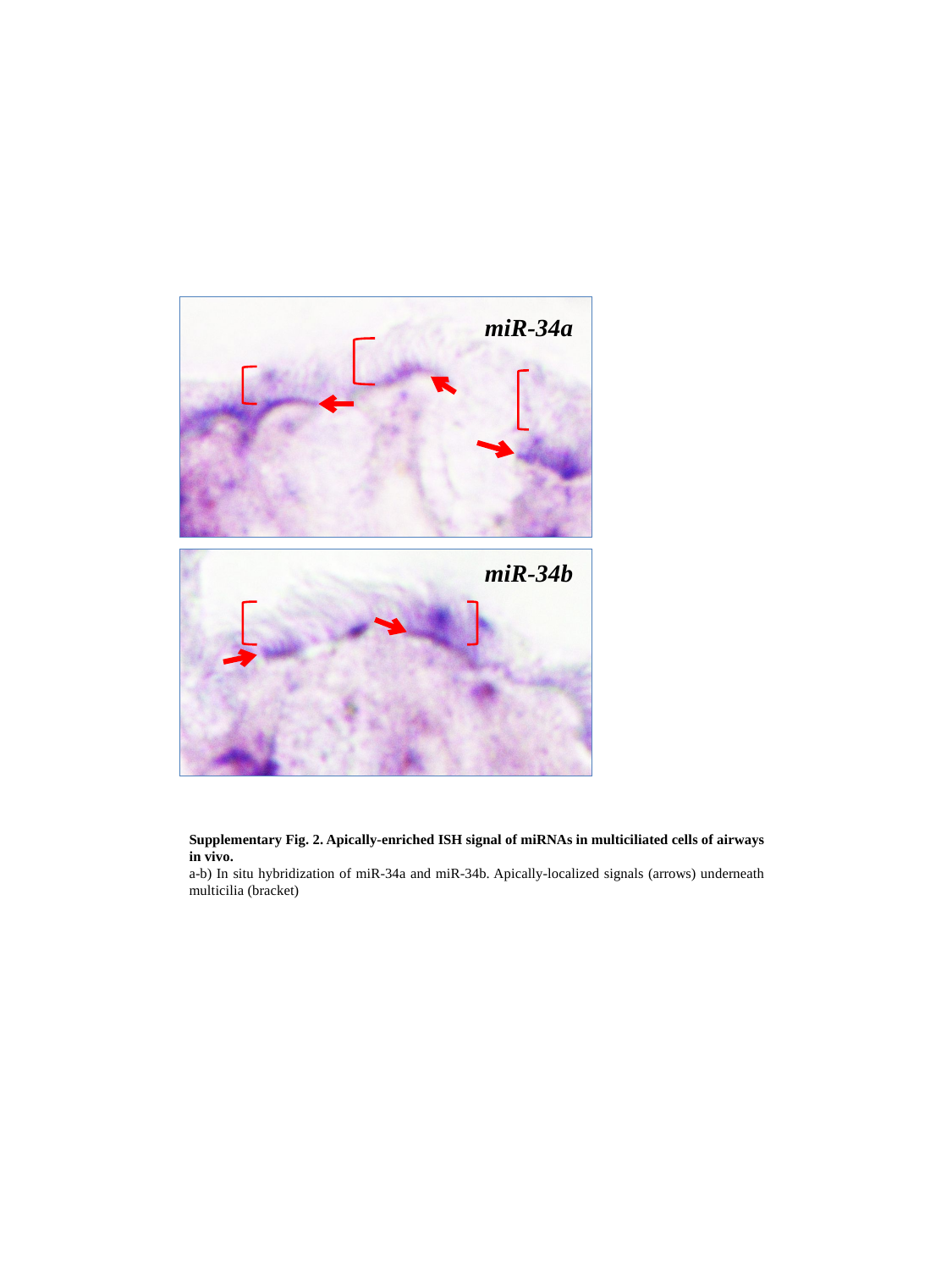

miR-34a
miR-34b
Supplementary Fig. 2. Apically-enriched ISH signal of miRNAs in multiciliated cells of airways in vivo.
a-b) In situ hybridization of miR-34a and miR-34b. Apically-localized signals (arrows) underneath multicilia (bracket)

## Slide 3
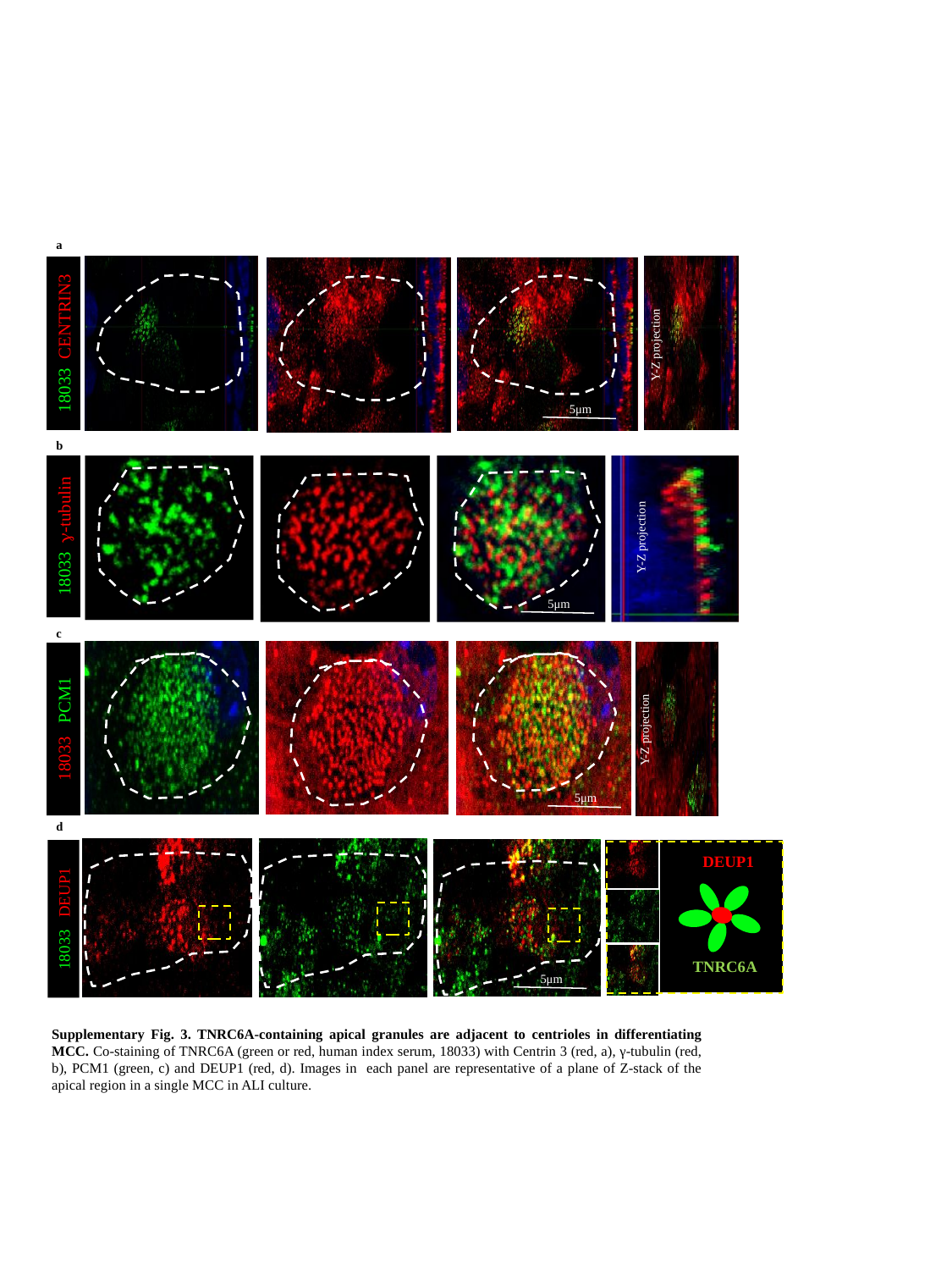

a
18033 CENTRIN3
Y-Z projection
5μm
b
18033 g-tubulin
Y-Z projection
d
5μm
c
18033 PCM1
Y-Z projection
5μm
d
DEUP1
TNRC6A
18033 DEUP1
5μm
Supplementary Fig. 3. TNRC6A-containing apical granules are adjacent to centrioles in differentiating MCC. Co-staining of TNRC6A (green or red, human index serum, 18033) with Centrin 3 (red, a), γ-tubulin (red, b), PCM1 (green, c) and DEUP1 (red, d). Images in each panel are representative of a plane of Z-stack of the apical region in a single MCC in ALI culture.

## Slide 4
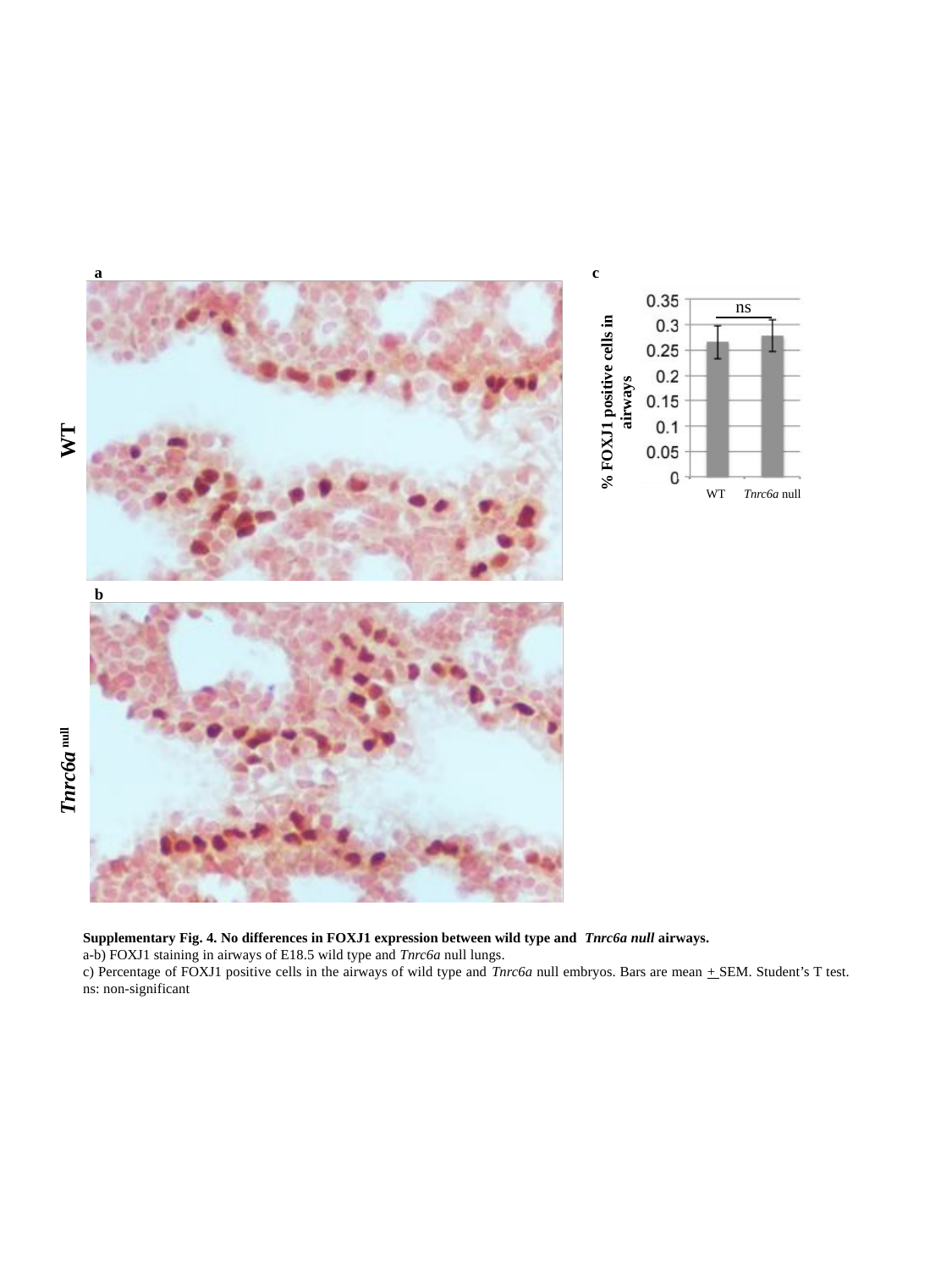

a
c
WT
Tnrc6a null
ns
% FOXJ1 positive cells in airways
WT
b
Tnrc6a null
Supplementary Fig. 4. No differences in FOXJ1 expression between wild type and Tnrc6a null airways.
a-b) FOXJ1 staining in airways of E18.5 wild type and Tnrc6a null lungs.
c) Percentage of FOXJ1 positive cells in the airways of wild type and Tnrc6a null embryos. Bars are mean + SEM. Student’s T test. ns: non-significant

## Slide 5
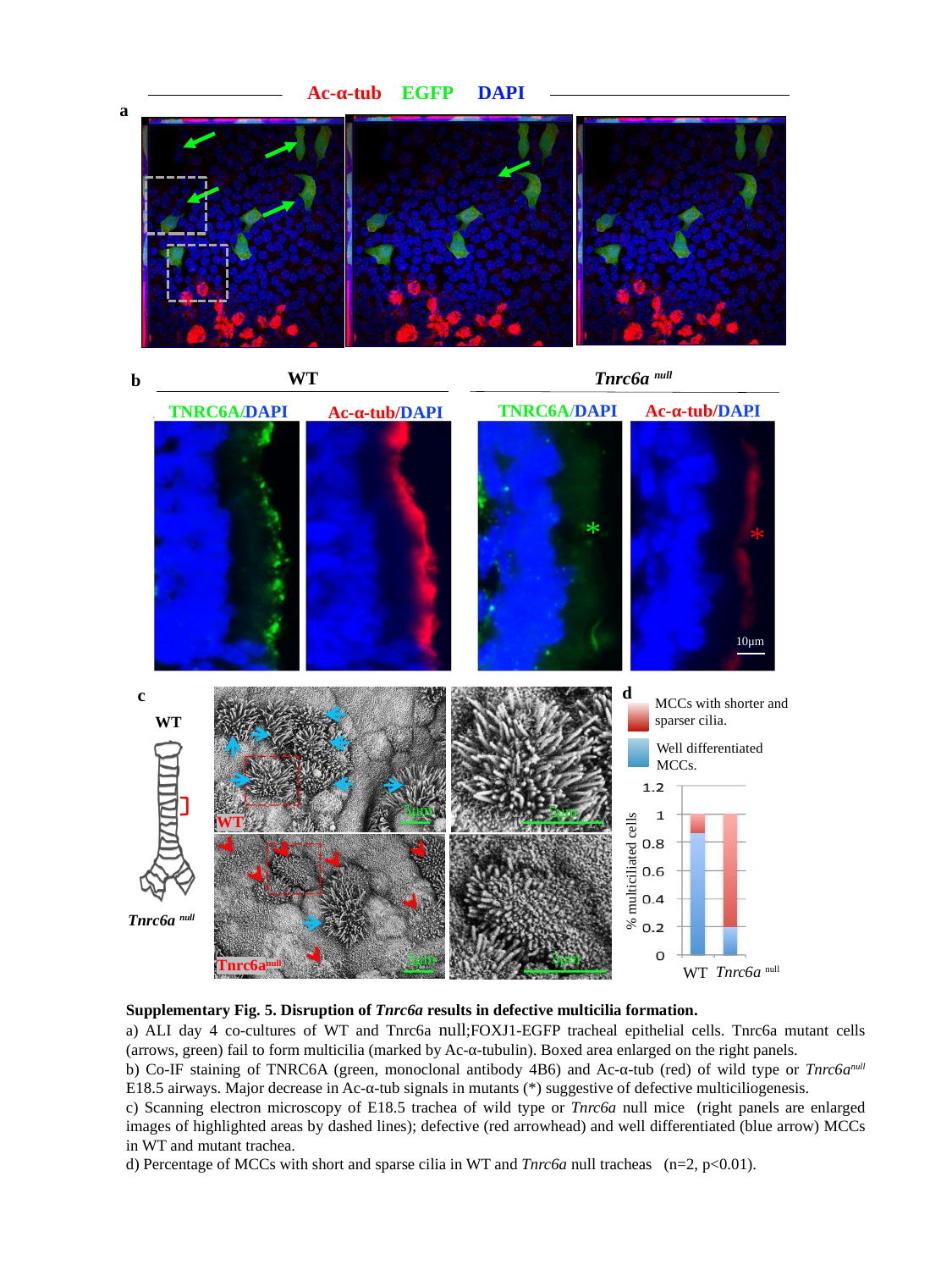

Ac-α-tub /EGFP /DAPI
a
Tnrc6a null
WT
b
TNRC6A/DAPI
Ac-α-tub/DAPI
TNRC6A/DAPI
Ac-α-tub/DAPI
10μm
d
MCCs with shorter and sparser cilia.
Well differentiated
MCCs.
% multiciliated cells
 Tnrc6a null
WT
c
WT
5μm
5μm
WT
Tnrc6a null
5μm
5μm
Tnrc6anull
*
*
Supplementary Fig. 5. Disruption of Tnrc6a results in defective multicilia formation.
a) ALI day 4 co-cultures of WT and Tnrc6a null;FOXJ1-EGFP tracheal epithelial cells. Tnrc6a mutant cells (arrows, green) fail to form multicilia (marked by Ac-α-tubulin). Boxed area enlarged on the right panels.
b) Co-IF staining of TNRC6A (green, monoclonal antibody 4B6) and Ac-α-tub (red) of wild type or Tnrc6anull E18.5 airways. Major decrease in Ac-α-tub signals in mutants (*) suggestive of defective multiciliogenesis.
c) Scanning electron microscopy of E18.5 trachea of wild type or Tnrc6a null mice (right panels are enlarged images of highlighted areas by dashed lines); defective (red arrowhead) and well differentiated (blue arrow) MCCs in WT and mutant trachea.
d) Percentage of MCCs with short and sparse cilia in WT and Tnrc6a null tracheas (n=2, p<0.01).

## Slide 6
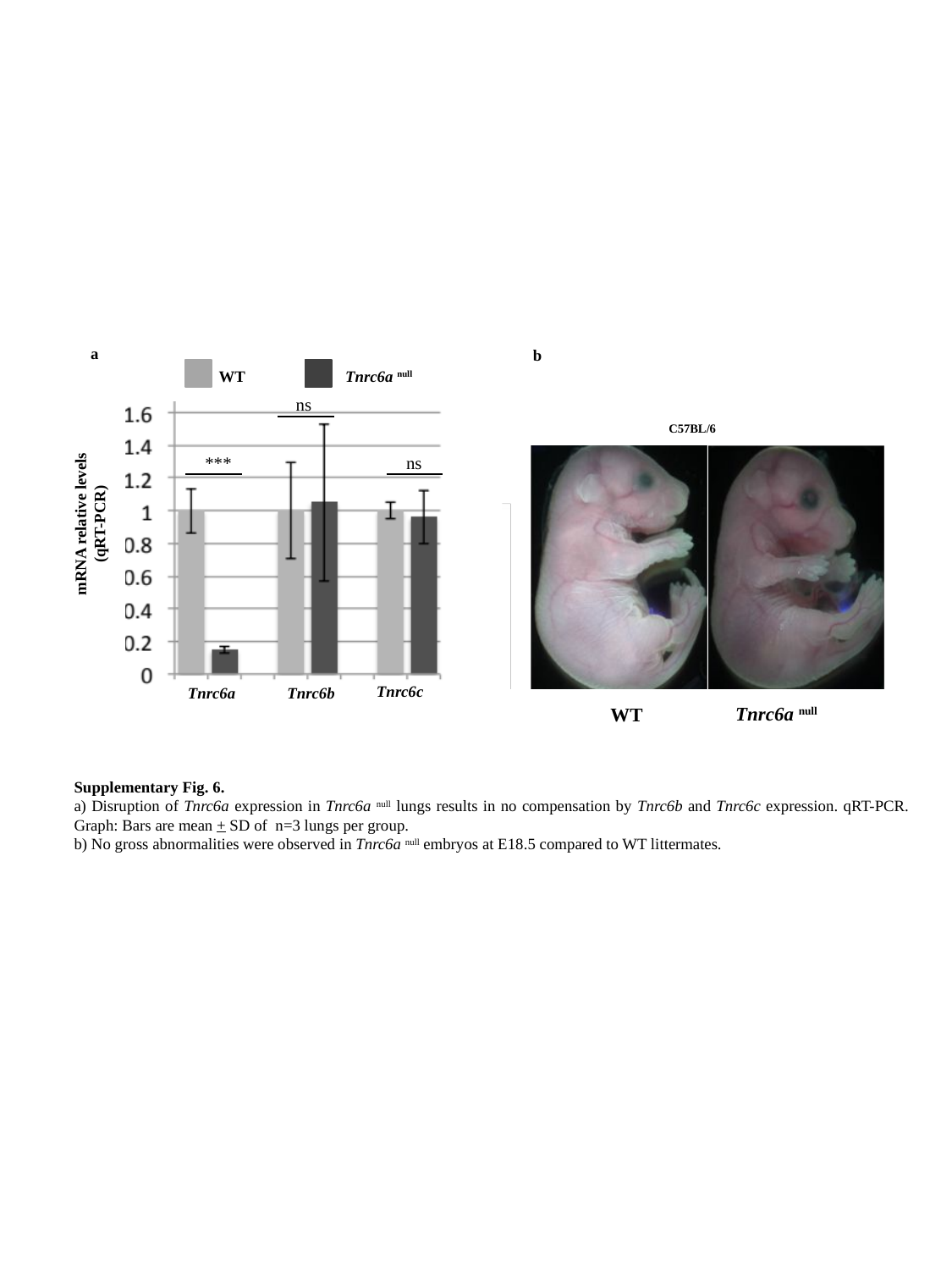

a
b
WT
Tnrc6a null
C57BL/6
mRNA relative levels
(qRT-PCR)
Tnrc6c
Tnrc6a
Tnrc6b
Tnrc6a null
WT
Supplementary Fig. 6.
a) Disruption of Tnrc6a expression in Tnrc6a null lungs results in no compensation by Tnrc6b and Tnrc6c expression. qRT-PCR. Graph: Bars are mean + SD of n=3 lungs per group.
b) No gross abnormalities were observed in Tnrc6a null embryos at E18.5 compared to WT littermates.
ns
***
ns

## Slide 7
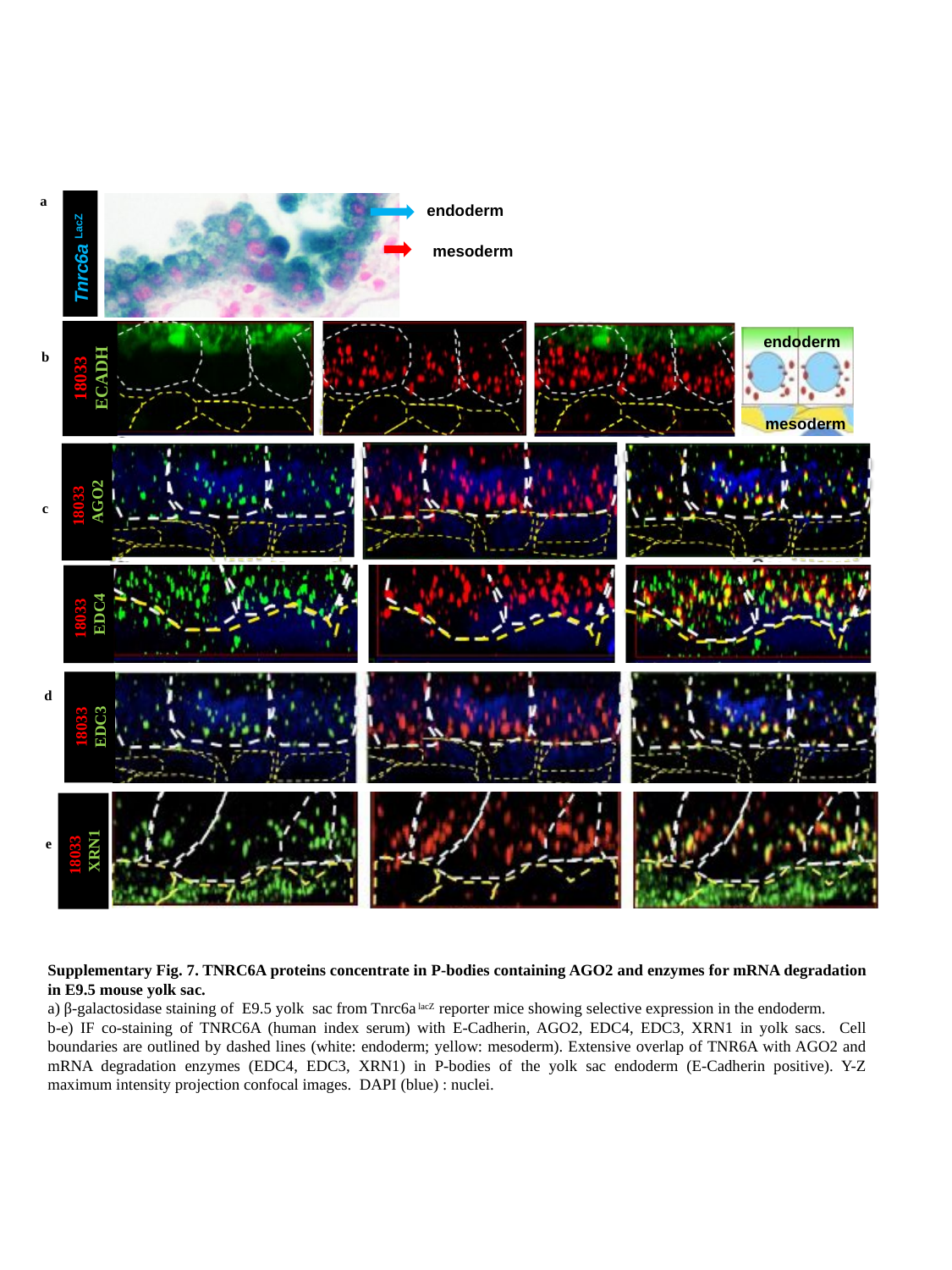

a
endoderm
mesoderm
Tnrc6a LacZ
18033 ECADH
18033 /
AGO2
18033 /
EDC4
18033
EDC3
18033 /
XRN1
endoderm
b
mesoderm
c
d
e
Supplementary Fig. 7. TNRC6A proteins concentrate in P-bodies containing AGO2 and enzymes for mRNA degradation in E9.5 mouse yolk sac.
a) β-galactosidase staining of E9.5 yolk sac from Tnrc6a lacZ reporter mice showing selective expression in the endoderm.
b-e) IF co-staining of TNRC6A (human index serum) with E-Cadherin, AGO2, EDC4, EDC3, XRN1 in yolk sacs. Cell boundaries are outlined by dashed lines (white: endoderm; yellow: mesoderm). Extensive overlap of TNR6A with AGO2 and mRNA degradation enzymes (EDC4, EDC3, XRN1) in P-bodies of the yolk sac endoderm (E-Cadherin positive). Y-Z maximum intensity projection confocal images. DAPI (blue) : nuclei.

## Slide 8
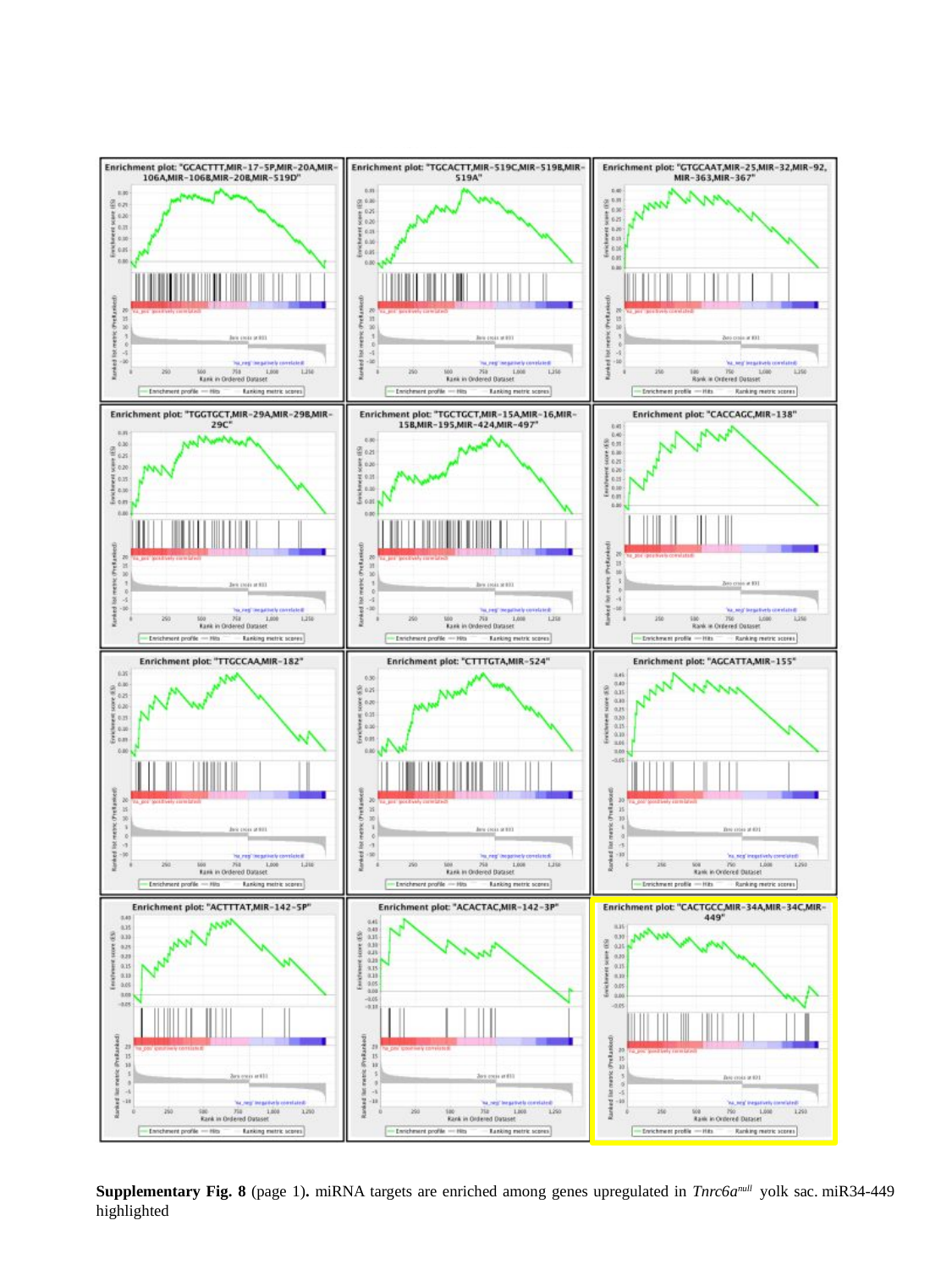

Supplementary Fig. 8 (page 1). miRNA targets are enriched among genes upregulated in Tnrc6anull yolk sac. miR34-449 highlighted

## Slide 9
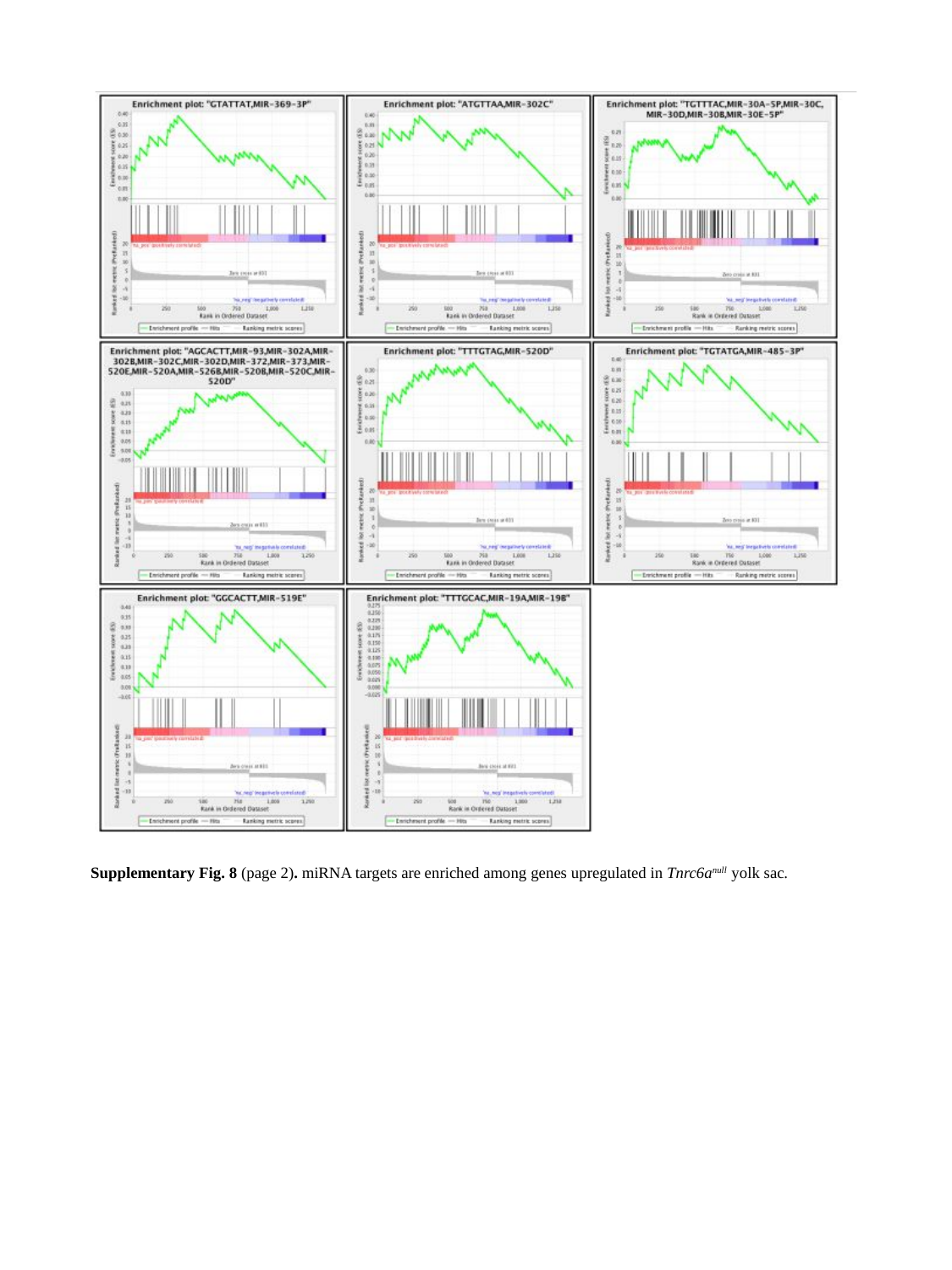

Supplementary Fig. 8 (page 2). miRNA targets are enriched among genes upregulated in Tnrc6anull yolk sac.

## Slide 10
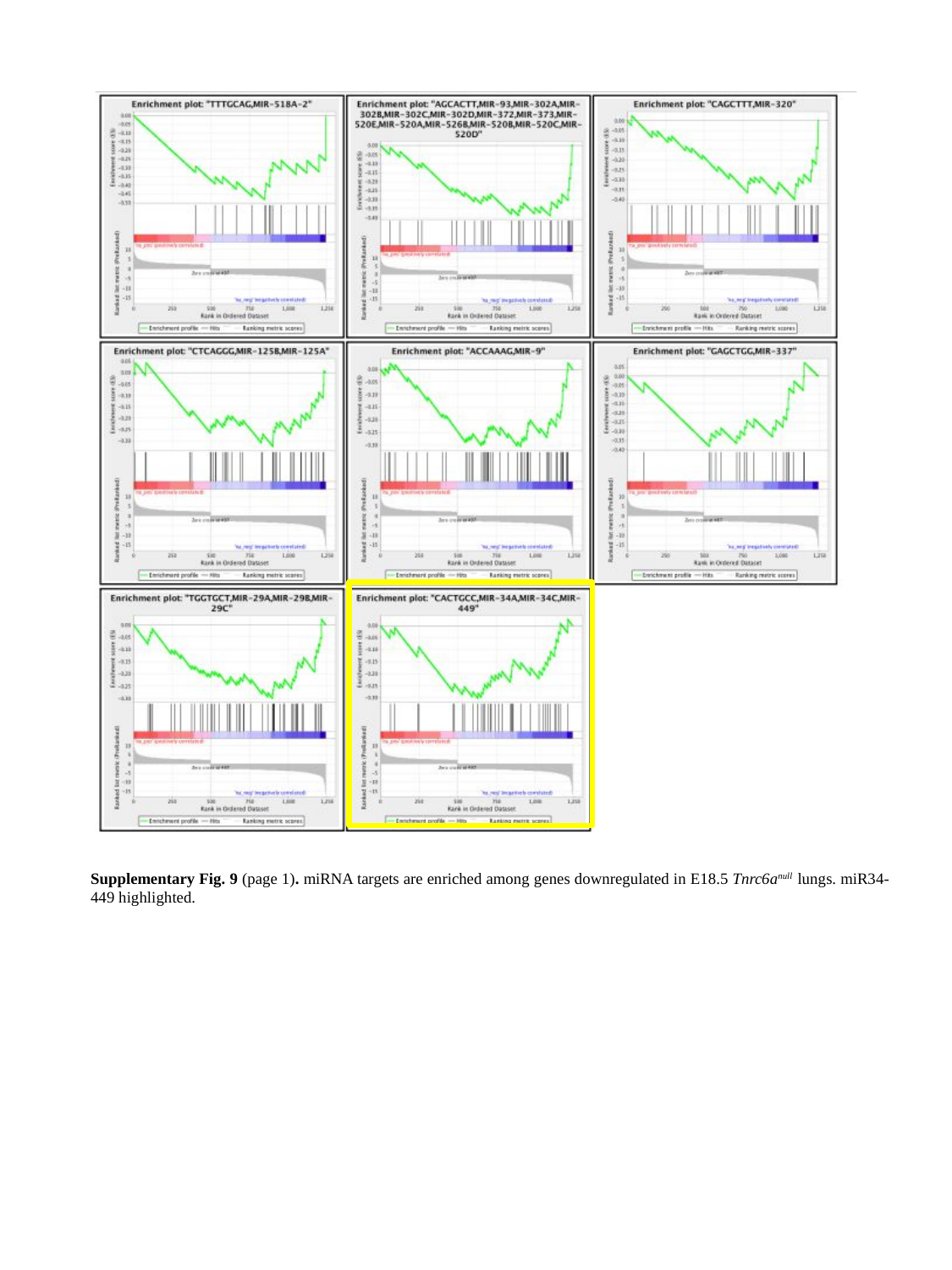

Supplementary Fig. 9 (page 1). miRNA targets are enriched among genes downregulated in E18.5 Tnrc6anull lungs. miR34-449 highlighted.

## Slide 11
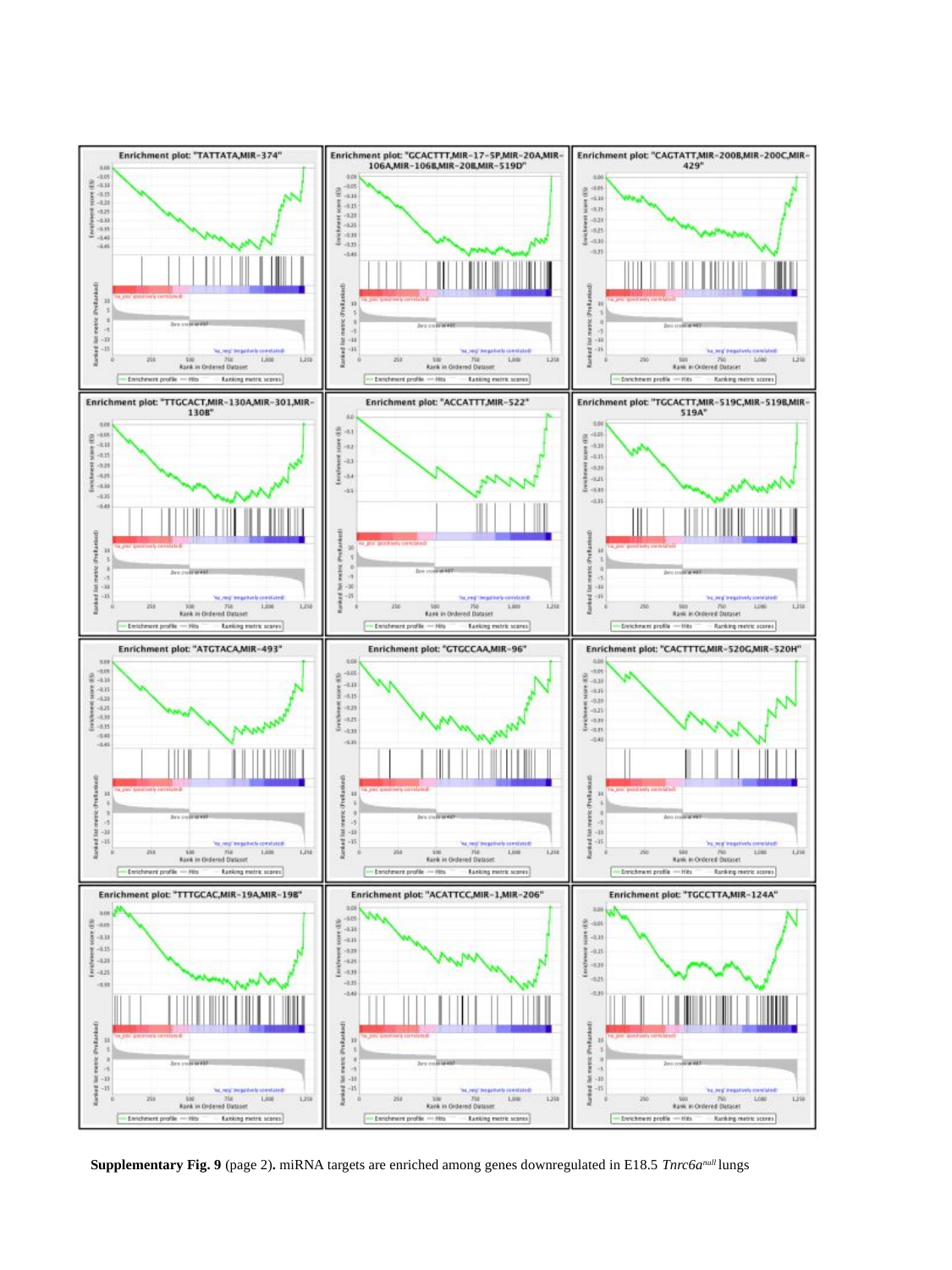

Supplementary Fig. 9 (page 2). miRNA targets are enriched among genes downregulated in E18.5 Tnrc6anull lungs

## Slide 12
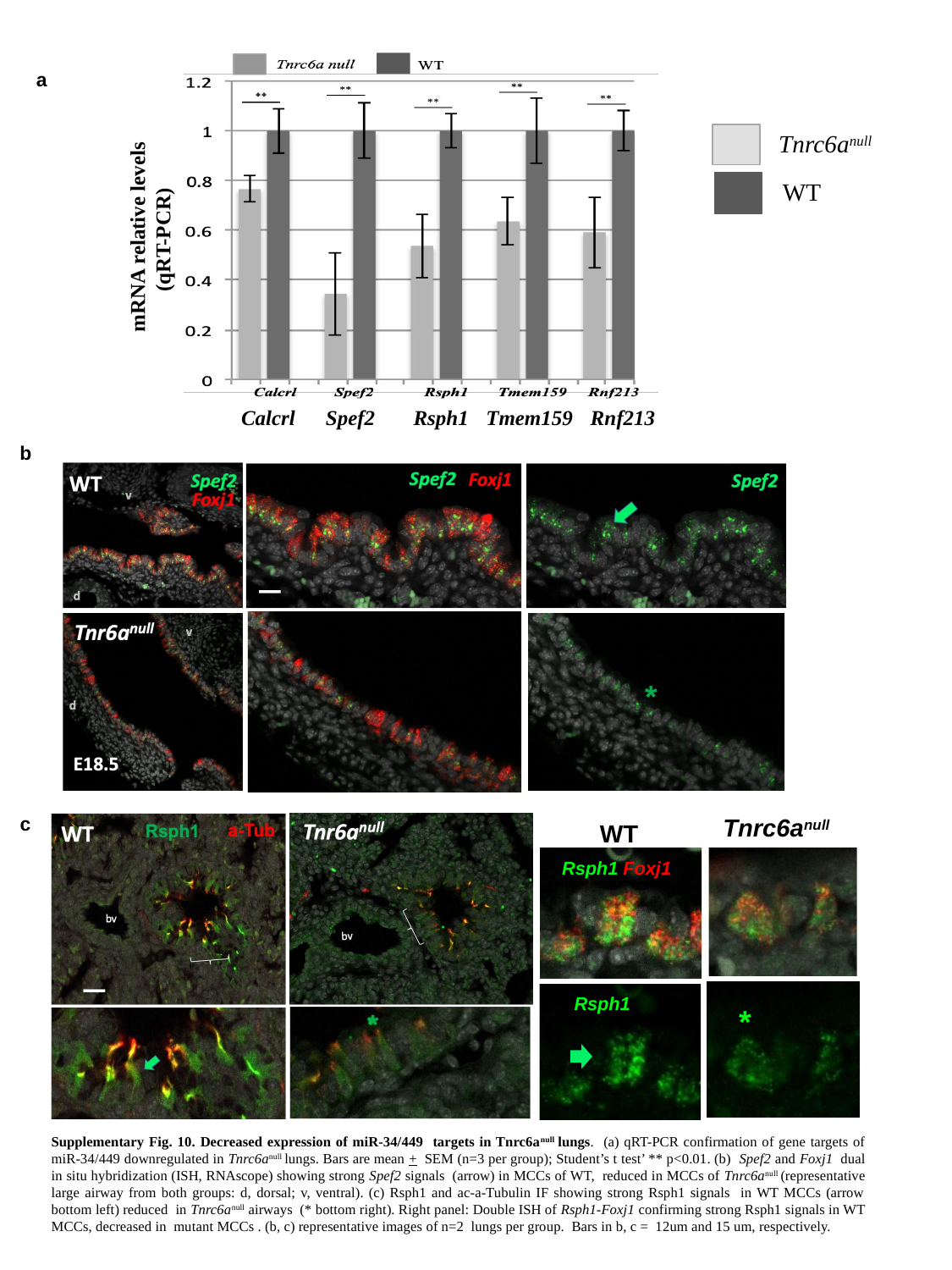

Calcrl
Spef2
Rsph1
Tmem159
Rnf213
a
Tnrc6anull
WT
 mRNA relative levels
(qRT-PCR)
b
I
c
Tnrc6anull
WT
Rsph1 Foxj1
Rsph1
*
I
Supplementary Fig. 10. Decreased expression of miR-34/449 targets in Tnrc6anull lungs. (a) qRT-PCR confirmation of gene targets of miR-34/449 downregulated in Tnrc6anull lungs. Bars are mean + SEM (n=3 per group); Student’s t test’ ** p<0.01. (b) Spef2 and Foxj1 dual in situ hybridization (ISH, RNAscope) showing strong Spef2 signals (arrow) in MCCs of WT, reduced in MCCs of Tnrc6anull (representative large airway from both groups: d, dorsal; v, ventral). (c) Rsph1 and ac-a-Tubulin IF showing strong Rsph1 signals in WT MCCs (arrow bottom left) reduced in Tnrc6anull airways (* bottom right). Right panel: Double ISH of Rsph1-Foxj1 confirming strong Rsph1 signals in WT MCCs, decreased in mutant MCCs . (b, c) representative images of n=2 lungs per group. Bars in b, c = 12um and 15 um, respectively.

## Slide 13
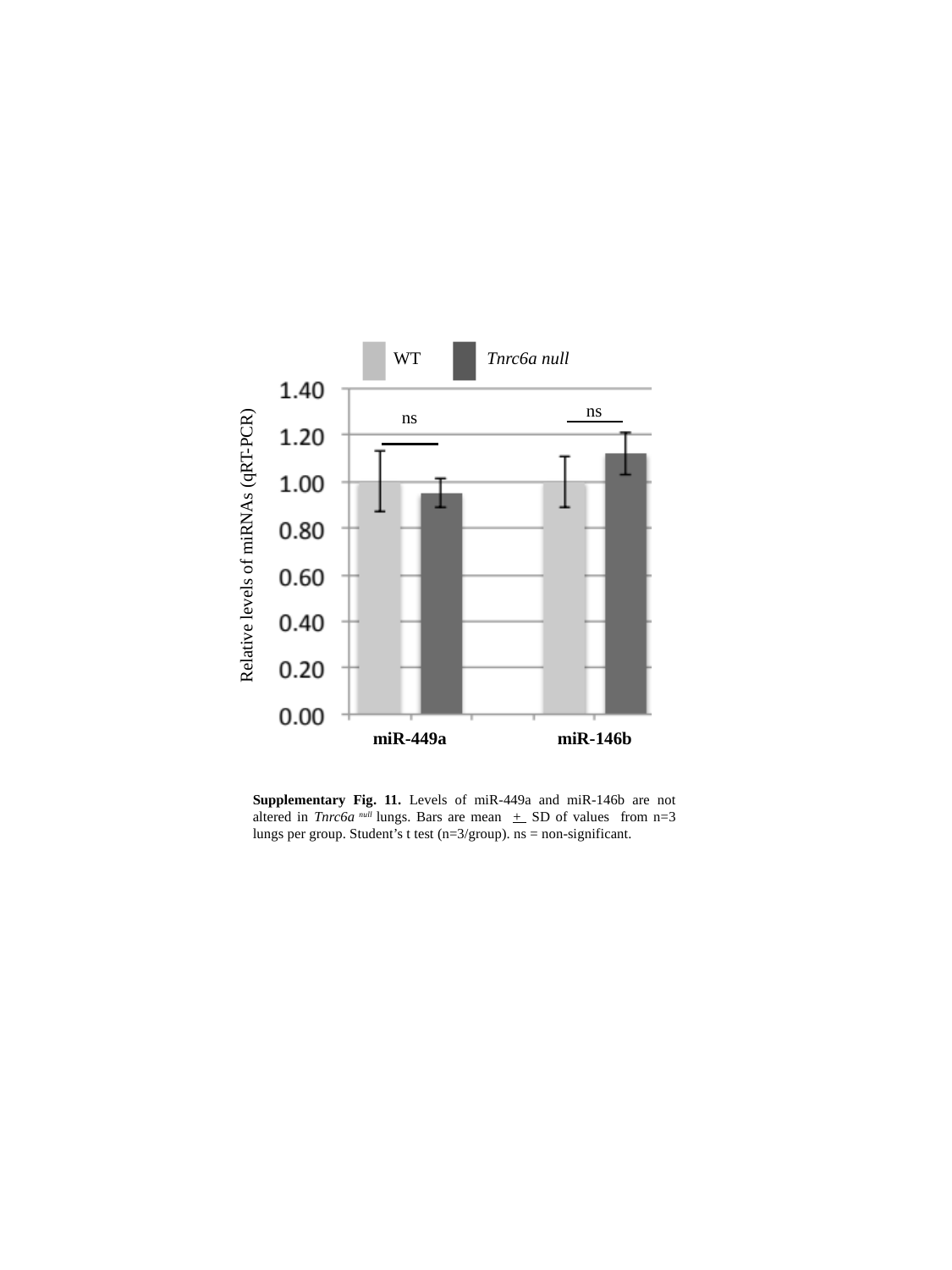

WT
Tnrc6a null
ns
ns
Relative levels of miRNAs (qRT-PCR)
miR-449a
miR-146b
Supplementary Fig. 11. Levels of miR-449a and miR-146b are not altered in Tnrc6a null lungs. Bars are mean + SD of values from n=3 lungs per group. Student’s t test (n=3/group). ns = non-significant.

## Slide 14
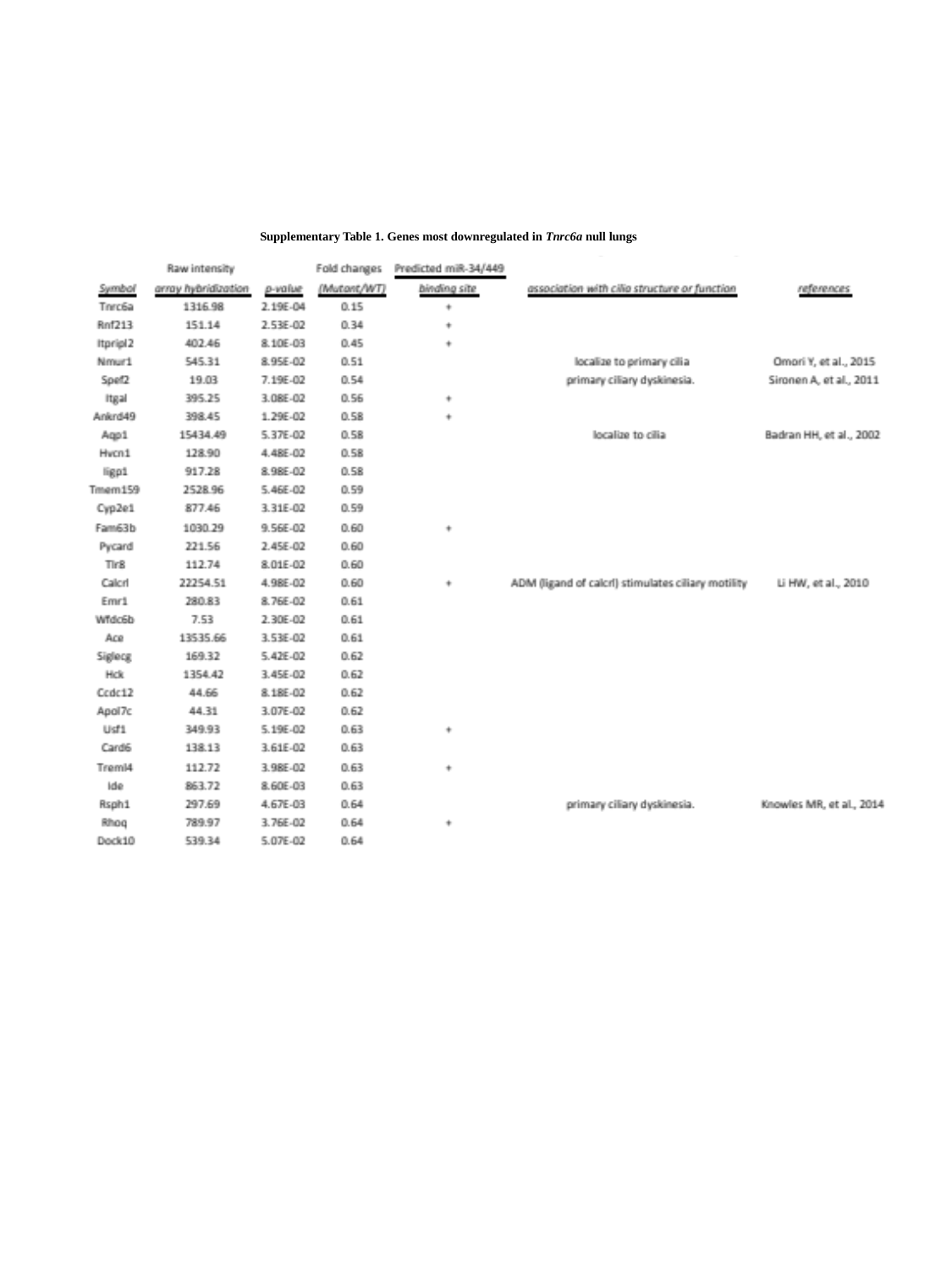

Supplementary Table 1. Genes most downregulated in Tnrc6a null lungs
